# Impact of Combined Hypertension and Diabetes on the Prevalence of Disability in Brazilian Older People—Evidence from Population Studies in 2013 and 2019

**DOI:** 10.3390/ijerph22071157

**Published:** 2025-07-21

**Authors:** Rafaela Gonçalves Ribeiro-Lucas, Barbara Niegia Garcia de Goulart, Patricia Klarmann Ziegelmann

**Affiliations:** 1 Postgraduate Program in Cardiology and Cardiovascular Sciences, Universidade Federal do Rio Grande do Sul (UFRGS), Porto Alegre 90610-264, Brazil; rafaelaglribeiro@gmail.com (R.G.R.-L.); patricia.ziegelmann@ufrgs.br (P.K.Z.); 2 Postgraduate Program in Epidemiology, Universidade Federal do Rio Grande do Sul (UFRGS), Porto Alegre 90035-003, Brazil

**Keywords:** aged, chronic disease, disability studies

## Abstract

Disability in basic and instrumental activities of daily living (BADL and IADL) reflects functional decline in older adults and can be associated with chronic conditions like type 2 diabetes (T2DM) and hypertension (SAH). This cross-sectional study utilized data from the 2013 and 2019 Brazilian National Health Surveys to investigate the associations between T2DM, SAH, and disability levels. Exposures were self-reported diagnoses and outcomes were classified as independent, moderate, or severe. Multivariable Poisson regression models, with robust variance estimates, estimated adjusted prevalence ratios (PRa), accounting for sociodemographic variables and the survey design. In 2013, the absence of diabetes and hypertension was associated with a lower prevalence (PRa = 0.70; 95% CI: 0.58–0.85) of moderate disability in BADL when compared with the presence of only one of the conditions. On the other hand, the coexistence of T2DM and SAH was associated with a higher prevalence (PRa = 1.39; 95% CI: 1.01–1.91). A similar result was found in 2019 with the addition that coexistence was also associated with a higher prevalence of severe disability in BADLs (PRa = 1.82; 95% CI: 1.59–2.07). For IADL, the absence of T2DM and SAH was associated with a lower prevalence of severe disability in 2013 and 2019 and a lower prevalence of moderate disability only in 2019. However, coexistence showed a higher prevalence in both degrees of disability and both years of the survey. These findings highlight the impact of T2DM and SAH on disability in older people. Therefore, it is crucial to develop targeted strategies for vulnerable subgroups to enhance functional independence in aging populations.

## 1. Introduction

Chronic noncommunicable diseases (NCDs), such as type 2 diabetes mellitus (T2DM) and systemic arterial hypertension (SAH), represent a major challenge to global public health. According to the Global Burden of Disease (GBD) study, these conditions are among the leading causes of morbidity and mortality worldwide. SAH is the foremost risk factor for cardiovascular diseases, contributing substantially to global mortality and disability-adjusted life years (DALYs) [1]. T2DM also ranks among the primary conditions associated with high rates of mortality and disability [2]. Both T2DM and SAH are key risk factors for cardiovascular diseases, which remain the leading cause of death in Brazil [3]. This global scenario places a significant burden on healthcare systems, increasing the demand for outpatient care, pharmacological treatment, and hospitalization [4].

According to the Brazilian National Health Survey (2019 PNS), the prevalence of SAH increased with age, reaching 56.6% among people aged 65 to 74 and 62.1% among those aged 75 or over [5]. Likewise, the prevalence of T2DM was 21.9% for people aged 65 to 74. For those aged 75 or over, the percentage was 21.1% [5]. In Brazil, the impact of aging on functionality is significant, as approximately 9.5% (or 3.3 million) of people aged 60 or over reported functional limitations in performing activities of daily living [6]. The mechanisms underlying the relationship between SAH, T2DM, and disability involve several pathophysiological factors that increase cardiovascular events and mortality [7]. In the case of diabetes, disability can be attributed to chronic inflammation and its micro- and macrovascular complications, such as neuropathy, peripheral vascular disease, retinopathy, and stroke, which directly compromise the mobility and functionality of individuals [8].

In a systematic review with meta-analysis, which aimed to estimate the magnitude of the association between diabetes/prediabetes and disability in adults, it was found that diabetes was associated with a high prevalence of both basic activities of daily living (BADL) disability (OR = 1.82; 95% CI 1.63–2.04) and instrumental activities of daily living (IADL) disability (OR = 1.65; 95% CI 1.55–1.74) [9]. On the other hand, a representative population study of the elderly Chinese population with SAH shows that the prevalence of disability for BADL or IADL was observed to increase proportionally with the increase in the number of comorbidities (OR = 1.76; 95% CI 1.321–2.367 for one comorbidity and OR = 3.66; 95% CI 2.819–4.763 for two comorbidities) [10].

Reis-Júnior and collaborators (2024), using data from the Brazilian National Health Surveys (2013 PNS), found that the prevalence of BADL disability is higher among the elderly (>=60 years) with SAH when compared to those without (PR = 1.60; 95% CI 1.43–1.79), as well as there being a greater prevalence of IADL disability (PR = 1.41; 95% CI 1.28–1.54) [11]. However, to date, we have not found studies evaluating the specific concomitant combination of hypertension and diabetes associated with functional disability, leaving it unclear how this combination affects the functional capacity. Thus, this study aimed to evaluate whether the combination of diabetes and hypertension is a factor associated with BADL and IADL disabilities in the population of non-institutionalized older Brazilians, using data from two representative surveys of the Brazilian population (2013 and 2019 PNS).

## 2. Materials and Methods

This study is based on data from the Brazilian National Health Survey (PNS) collected in 2013 and 2019. The PNS is an epidemiological surveillance initiative designed to monitor the health of the Brazilian population. This is a cross-sectional household survey study with complex sampling, conducted by the Oswaldo Cruz Foundation (Fiocruz, Rio de Janeiro, Brazil), the Brazilian Institute of Geography and Statistics (IBGE, Rio de Janeiro, Brazil), and the Ministry of Health (MS, Brasília, Brazil). The complex probabilistic cluster sampling plan with three stages ensures the national and regional representativeness of the survey [12].

Both editions of the PNS survey maintained a similar sampling design. To ensure comparability, the sample weights from 2013 were recalculated using updated population projections by sex and age, released by the IBGE in 2018. Small changes were made, including adjustments to household sample sizes and an expansion of the minimum age range for individual interviews [5]. The PNS questionnaire was divided into three parts in both editions: the first collects information about the household; the second gathers data on all household residents; and the third addresses individual questions from a randomly selected resident. Changes have been made to some questions and/or response categories. The data collection in both surveys was carried out using mobile devices. Additionally, the PNS questionnaire underwent cognitive test evaluations and a pilot study. Also, they were duly reviewed and approved by the National Research Ethics Committee (CONEP/CNS), as per opinions No. 328.159 issued on 8 July 2013 and No. 3.529.376 on 23 August 2019 [13]. All participants signed informed consent forms in two copies. More details about the PNS methodology are described in specific articles [12,13,14,15].

For this study, we included data from elderly individuals aged 60 or over who responded to the third part of the questionnaire in both editions of the PNS. Participants who did not answer the question about a self-reported medical diagnosis of T2DM and SAH were excluded from the analysis. All data used were anonymized and publicly available. Therefore, according to the National Health Council Resolution 466/12, this study does not require Ethics Committee approval. This manuscript will be reported based on the STROBE Statement for cross-sectional studies [16].

The exposure of interest is the self-reported medical diagnosis of T2DM and SAH. The variable created has three categories: only T2DM or SAH, both, or neither. This study focused on two main outcomes: Basic Activities of Daily Living (BADL) and Instrumental Activities of Daily Living (IADL) disabilities. For the BADL assessment, individuals were queried on their ability to perform tasks such as eating, bathing, using the toilet, dressing, walking between rooms, lying down, and sitting alone. Responses ranged from “No difficulty” to “Unable”, categorized into binary variables (0 for no difficulty, 1 for any degree of difficulty). A BADL score was derived by summing these variables, and then the subjects were categorized as independent (6 points), moderate (3–5 points), or severe (0–2 points). To align with the original scale, a “transfer” variable was created by combining “lying down” and “sitting” responses.

For the IADL assessment, individuals were asked about managing tasks such as shopping, managing finances, taking medication, attending medical appointments, and using transportation alone. An IADL score was similarly created with cut-offs of independent (5 points), moderate (3–4 points), or severe (0–2 points). Notably, these scales were adapted from the Katz scale [17], modified in the PNS to assess difficulty rather than dependence.

Further modifications included the adaptation of the IADL question regarding medication management between the 2013 and 2019 PNS surveys, addressing swallowing, scheduling, and remembering medication. NA values were considered “no difficulty”, following consultations with aging specialists and the Ministry of Health’s technical area on Elderly Health.

The response categories of the Katz and Lawton scales [17] were aligned with the Washington Group Short Set on Functioning (WG-SS), designed to standardize functional limitation assessments globally. This framework, beginning with “Do you have difficulty in...”, offers four response options: no difficulty, some difficulty, a lot of difficulty, and cannot do at all. This standardization supports international and longitudinal comparisons, vital for monitoring health disparities [18].

The classification of functional disability levels (independent, moderate dependency, and severe dependency) was based on the Medication and Quality of Life in Frail Older Persons (MedQoL) Research Group’s methodology, which established cut-off points through a systematic review and expert panel consensus [19]

Based on the specialized literature on the subject [20,21,22], the following variables were chosen for the adjusted analyses: sex, age group (60–69, 70–79, 80–89, and 90+), region of the country, marital status, education level, and ethnicity. Appendix A presents the operational definitions of those variables at the baseline, as well as the new categorizations (Table A1), along with the adherence of this study to the STROBE criteria (Table A2) and with the raw data (Table A3, Table A4, Table A5 and Table A6).

Data were described using absolute and relative frequencies. Prevalence was estimated using binomial and multinomial models. The contribution of TDM2 and SAH to disability was analyzed separately for BADL and IADL. The association between the exposure factor and each disability was assessed using the adjusted Poisson regression model with a robust estimation of the variance. The prevalence and prevalence ratios were weighted according to the complex sample plan of the PNS. Data importation, variable manipulation, data frame creation, statistical analyses, and data visualization were performed using R software, version R-4.4.2, and RStudio version 2024.12.0+467, utilizing the Comprehensive R Archive Network (CRAN) repository packages: readr, ggplot2, PNSIBGE, dplyr, tidyr, crosstable, survey, foreign, lmtest, sandwich, multcomp, and geobr.

## 3. Results

### 3.1. Sample Description

**Figure 1:**
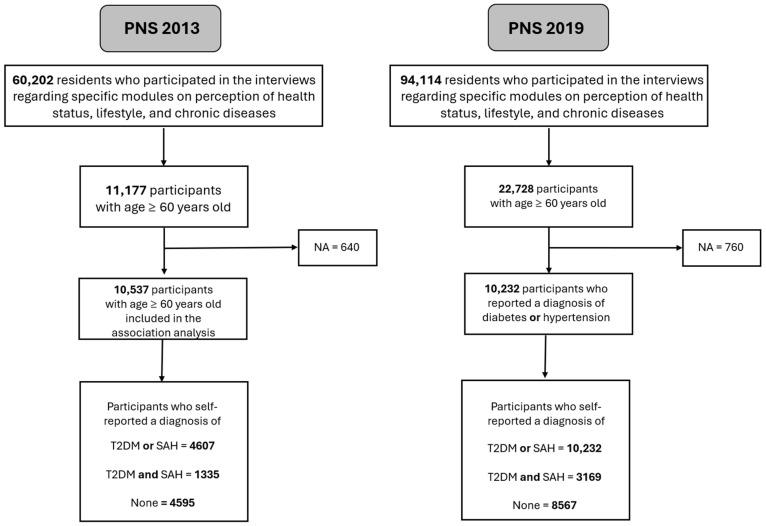
Flowchart of study participants, Brazilian National Health Survey, 2013 and 2019.

For the PNS 2013 analysis, individuals aged 18 years or older were included, totaling 60,202 respondents who answered the third individual questionnaire. After filtering the data to include only elderly individuals aged 60 years or older, 11,177 participants were identified and were considered to estimate the prevalence. Excluding individuals without answers to T2DM or SAH, 10,537 were considered for the association analysis. In the 2019 PNS edition, those numbers were 94,114; 22,728; and 21,968 (Figure 1).

### 3.2. Brazilian National Health Survey (2013 PNS) Sample Description

The sample was composed mostly of females (pw = 56.4%) who were black (pw = 44.8%), without a partner (pw = 46.5%), with elementary school as a minimum level of education (pw = 70.7%), a resident of an urban region (pw = 85.2%), and with a mean age of 69 years (SD 7.88). Among the participants, 4607 were diagnosed with hypertension or diabetes (pw 44.3%), 1335 with both conditions (pw = 13.6%), and 4595 (pw = 42.1%) without any of these diseases. Regarding BALD functionality, 9424 were independent (they had no difficulty performing activities), 1197 had moderate disability, and 556 had severe disability. In contrast, in the IADL, 7893 were independent (they had no difficulty performing activities), 1540 had moderate disability, and 1744 had severe disability (Table 1).

### 3.3. Brazilian National Health Survey (PNS 2019) Sample Description

The 2019 sample shows similar characteristics: the majority was female (pw = 56.7%), black (pw = 47.7%), without a partner (pw = 49.3%), with elementary school as the level of education (pw = 63.3%), residents of an urban region (pw = 85.5%), and with a mean age of 70 years (SD 7.85) (Table 2). Among the participants, 10,232 were diagnosed with hypertension or diabetes (47.5%), 3169 with both conditions (15.2%), and 8567 (37.3%) without any of these diseases. About BALD functionality, 18,141 were independent (they had no difficulty performing activities), 3040 had moderate disability, and 1547 had severe disability. On the other hand, for the IADL, 2967 were independent (they had no difficulty performing activities), 14,354 had moderate disability, and 5407 had severe disability.

### 3.4. Disability Prevalences

**Figure 2:**
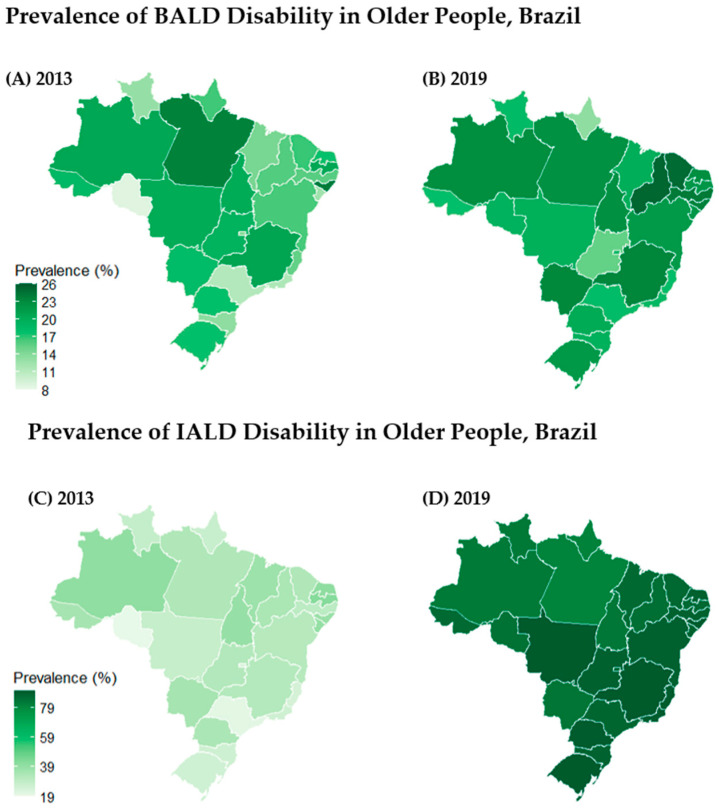
Map of the prevalence of BADL and IALD disability in older people in Brazil, according to data from the Brazilian National Health Survey, 2013 and 2019. Source: Brazilian National Health Survey, 2013 and 2019|Graphics: Drawn by the authors.

Figure 2 illustrates the spatial distribution by the state of the prevalence of BADL and IADL among older adults in Brazil for both 2013 and 2019. While disability in BADLs ranged from 15% to 25% (Figure 2, map A), disability in IADL presented a higher range, between 20% and 40% (Figure 2, map C).

Geographically, it is observed that the states with the highest disability prevalence of BADL also tend to have a high prevalence of IADL. As illustrated in maps A and B, disability in BADL ranged from 12% to 24%. In 2013, the state with the lowest prevalence was Rondônia, at 8.72%, and the state with the highest prevalence was Alagoas, at 24.01%.

In 2019, the state with the lowest prevalence of BALD disability was Amapá (13.38%) and the highest was Piauí (25.25%). In 2013, regarding the prevalence of disability in IADL, the lowest prevalence was observed in the southern region at 24.92%, followed by the southeast region at 25.64%, the midwest at 28.98%, the north at 31.46%, and the northeast at 34.93%. Likewise, in BALD, the state with the lowest prevalence was Rondônia, at 19.18%, and the state with the highest prevalence was Paraíba, at 42.04%. In 2019, the region with the lowest prevalence of IADL disability was the north, at 83.83%, followed by the midwest (87.26%), northeast (87.59%), southeast (88.33%), and south (89.40%). In 2019, the state with the lowest prevalence of IADL disability was Pará (80.32%) and the highest was Rio Grande do Sul (90.38%).

### 3.5. Association Between T2DM/SAH and BADL/IADL

In 2013, the absence of diabetes and hypertension was associated with a lower prevalence (PRa = 0.70; 95% CI: 0.58–0.85) of moderate disability in BADL when compared with the presence of only one of the conditions (Table 3). On the other hand, the coexistence of T2DM and SAH was associated with a higher prevalence (PRa = 1.39; 95% CI: 1.01–1.91). A similar result was found in 2019 with the addition that coexistence was also associated with a higher prevalence of severe disability in BADL (PRa = 1.82; 95% CI: 1.59–2.07) (Table 3). For IADL, the absence of T2DM and SAH was associated with a lower prevalence of severe disability in 2013 (PRa = 0.79; 95% CI: 0.69–0.90) and 2019 (PRa = 0.77; 95% CI: 0.73–0.81) and a lower prevalence of moderate disability only in 2019 (PRa = 0.76; 95% CI: 0.75–0.77) (Table 4).

## 4. Discussion

Our findings demonstrate that the absence of diabetes and hypertension plays a protective role in preserving functional independence among older adults in Brazil. Compared to individuals with only one chronic disease (diabetes or hypertension), those without either condition consistently showed a lower prevalence of functional disability, especially in IADL. On the other hand, individuals with both diabetes and hypertension combined experienced higher prevalence rates of BADL disability, moderate in 2013 and severe in 2019, highlighting the burden of multimorbidity in this population. Furthermore, the spatial distribution of disability prevalence reveals a consistent pattern of geographic disparity, particularly in the north and northeast regions of Brazil. These regions not only exhibited the highest prevalence of BADL disability in both years, but also showed a marked increase in IADL disability, with several states approaching the upper limit of the prevalence scale.

Associations between T2DM and hypertension are expected since they share several similar pathophysiological mechanisms, including the inadequate activation of the renin–angiotensin–aldosterone system, oxidative stress caused by the excessive production of reactive oxygen species, inflammatory processes, impaired insulin-mediated vasodilation, the increased activation of the sympathetic nervous system, dysfunction of innate and adaptive immune responses, and abnormalities in renal sodium handling [20].

Our results, along with previous findings in Brazilian populations [11,21,22,23,24,25,26,27] and in other populations worldwide [28,29,30,31,32], reflect the understanding that aging is a progressive process that leads to a decline in the physiological function in all organic systems and, together with the presence of chronic diseases such as diabetes and hypertension, can significantly impair essential daily activities of older adults [33], including limitations in walking, standing, sitting, or lifting objects due to diabetic neuropathy, infections, amputations, or cardiovascular diseases [34].

Although our study is not suitable for investigating causality, our results are in line with a recent Mendelian Randomization study that demonstrated that frailty doubles the chances of developing T2DM, supporting the hypothesis of a bidirectional causal relationship between T2DM and frailty [35].

The biggest strength of this study is its representative population base, as the data were extracted from the Brazilian National Health Survey of 2013 and 2019. The PNS is a comprehensive national survey driven by the IBGE in partnership with the Ministry of Health, using probabilistic complex sampling to ensure the representation of the Brazilian population. Using two versions of the same survey allowed for a temporal analysis of health conditions, identifying trends and changes in prevalence over the period evaluated. Thus, the findings reflect the country’s epidemiological scenario with high external validity. Another strength of this study is the standardization of data collection methods, ensuring comparability between the periods analyzed. The PNS uses structured questionnaires administered by trained interviewers, reducing measurement bias and ensuring the quality of the information. The inclusion of sociodemographic, behavioral, and health service access data allows for the appropriate adjustment of the analysis statistics, reducing potential confounding factors and strengthening inferences about the determinants of hypertension, diabetes, and disabilities.

The limitations of our study include the presentation of small sample sizes in some categories, especially among older people (90+), which may introduce survival bias, as well as a small sample size in the “other races” category. Added to this, other limitations include the formulation of the exposition, as only the self-report variable diagnosis was used to consider a case. There is evidence in the literature that the prevalence of diabetes can change dramatically when using other criteria points, such as considering the values of hemoglobin laboratory tests to be glycated and using the exclusion variable for the diagnosis of gestational diabetes [36].

In the context of our study, survivorship bias may have led to an underestimation of both the prevalence and the functional impact of diabetes and hypertension. This occurs because the study population consists only of individuals who survived into older age and were available to participate in the survey. As a result, those who experienced more severe forms of the disease, especially individuals with early-onset diabetes or major complications such as amputations, myocardial infarction, or stroke, may have died prematurely and are, therefore, not represented in the sample. Consequently, the associations observed may reflect a subset of individuals with less severe or better-controlled disease, leading to attenuated estimates of risk and an under-representation of the true burden of disability attributable to these conditions.

Additionally, this bias may help explain the decreasing prevalence ratios with advancing age observed in our data. The oldest age groups may appear “healthier,” not necessarily because they are unaffected, but because only the healthiest among them have survived, while those more severely impacted by chronic diseases were systematically excluded by mortality. In Picon and collaborators’ study, they have shown that there are disparities in the prevalence of hypertension in different outlines, such as national and telephone surveys [37]; therefore, as our study is a national household survey, this may be related to an underestimation of the prevalence evaluated.

The results of this study contribute to a growing body of evidence highlighting the functional repercussions of chronic conditions such as diabetes and hypertension among older adults in Brazil. These findings reinforce the need for integrated public health strategies that move beyond disease control to encompass functional health and autonomy as key outcomes in aging populations. In this context, policies aimed at early detection, comprehensive care, and multidisciplinary management of chronic diseases become essential to slow the progression of disability. Additionally, the disproportionate burden of functional decline among socioeconomically and racially marginalized groups underscores the urgency of addressing health disparities. Developing targeted, equity-oriented interventions that prioritize accessibility and continuity of care may mitigate long-term consequences and improve the quality of life of vulnerable older adults. By recognizing functionality as a central component of healthy aging, public health actions can better align with the complex and multidimensional needs of this population [38].

## 5. Conclusions

This population-based study with adults living in Brazil of 60 years and older demonstrated that the combination of chronic conditions, such as diabetes and hypertension, significantly impacts the functional capacity of older individuals, with varying magnitudes depending on the different levels of functional commitment (BALD and IADL). Unlike previous research on Brazilians, which does not explore the specific combination of diabetes and hypertension comorbidities from a population perspective. This study highlights the relevance of this specific combination, demonstrating that its influence on disability in older populations occurs in a differentiated way, reinforcing the need to consider it in clinical and epidemiological research.

The comparison between BALD and IADL suggests that the loss of functionality occurs progressively, starting with difficulties in more complex activities and progressing to limitations in basic activities as frailty increases. This pattern reinforces the need for early interventions aimed at preserving independence in IADL to delay or prevent the progression of disability to BADL, which can result in greater dependence and an overload on health and social care systems.

The findings of this study have practical implications that extend beyond epidemiological observation. From a clinical standpoint, they support the growing recognition that older adults should be stratified not only by the presence of chronic diseases, but also by the severity of their functional limitations. This approach is especially relevant in geriatric medicine and rehabilitation, where multimorbidity and functional decline frequently coexist and demand individualized, multidimensional interventions. In public health surveillance, our results reinforce the need to monitor the functional impacts of diabetes and hypertension over time, informing resource allocation and the design of targeted programs for high-risk older adults. Furthermore, these findings align with a shift in disability paradigms from a purely medical model focused on disease to a more integrated perspective that incorporates the social model and the human rights model. This broader framework emphasizes autonomy, inclusion, and equity in health care for older adults and supports the development of policies that address functional limitations not only as medical outcomes, but also as social determinants of participation and well-being. Future research should explore how clinical care pathways and public health strategies can be restructured to reflect this multidimensional understanding of disability and aging, contributing to more just and effective care systems.

## Figures and Tables

**Table 1:** Prevalence of moderate and severe dependence in basic activities of daily living (BADLs) and instrumental activities of daily living (IADLs) according to sociodemographic characteristics in Brazilian older adults who participated in the Brazilian National Health Survey (2013 PNS).

	2013 PNS						
	BALD				IADL		
Variable	Total Population	Independent	Moderate	Severe	Independent	Moderate	Severe
	n = 11,177	n = 9424	n = 1197	n = 556	n = 7893	n = 1540	n = 1744
**Chronic Disease**							
Diabetes or Hypertension	4607 (44.3)	3794 (43.6)	567 (48.1)	246 (47.7)	3091 (42.9)	677 (46.8)	839 (48.5)
Both	1335 (13.6)	1021 (12.2)	208 (20.6)	106 (21.6)	786 (15.7)	242 (15.7)	307 (18.8)
None	4595 (42.1)	4055 (44.2)	366 (31.3)	174 (30.6)	3543 (18.8)	536 (37.4)	516 (32.7)
NA	640	554	56	30	473	85	82
**Age**							
60–69	6238 (56.6)	5590 (60.2)	512 (44.0)	136 (22.5)	5148 (66.2)	618 (39.5)	472 (25.6)
70–79	3441 (29.8)	2859 (29.3)	391 (32.2)	191 (34.3)	2220 (27.0)	631 (40.4)	590 (34.2)
80–89	1293 (11.6)	877 (9.2)	243 (19.6)	173 (32.9)	494 (6.3)	261 (17.9)	538 (30.8)
90+	205 (2.0)	98 (1.3)	51 (4.2)	56 (10.3)	31 (0.5)	30 (2.2)	144 (9.4)
**Sex**							
Female	6622 (56.4)	5508 (55.7)	754 (57.9)	360 (63.6)	4421 (53.8)	993 (59.0)	1208 (66.3)
Male	4555 (43.6)	3916 (44.3)	443 (42.1)	196 (36.4)	3472 (46.2)	574 (41.0)	536 (33.7)
**Race**							
White	5314 (53.8)	4477 (53.9)	569 (53.3)	268 (52.0)	3816 (54.5)	696 (53.4)	802 (50.5)
Black	5701 (44.8)	4807 (44.5)	615 (46.2)	279 (47.1)	3957 (43.8)	822 (45.5)	822 (48.7)
Others	160 (1.4)	139 (1.6)	12 (0.5)	9 (1.0)	119 (1.7)	21 (1.1)	20 (0.7)
NA	0	0	0	0	1	1	0
**Marital Status**							
With partner	4808 (53.5)	4207 (55.4)	444 (48.9)	753 (32.7)	3725 (58.0)	553 (45.6)	350 (39.0)
Without partner	6369 (46.5)	5217 (44.6)	157 (51.1)	399 (67.3)	4168 (42.0)	987 (54.4)	1214 (61.0)
**Education level**							
Until elementary school	7738 (70.7)	6337 (68.4)	953 (82.2)	448 (84.2)	4997 (64.6)	1,26 (84.4)	1481 (87.3)
High school or more	3439 (29.3)	3087 (31.6)	244 (17.8)	108 (84.2)	2896 (35.4)	280 (15.6)	263 (12.7)
**Region**							
Urban	8999 (085.2)	7575 (85.5)	953 (81.2)	471 (87.3)	6401 (86.2)	1211 (82.8)	1387 (82.5)
Rural	2178 (14.8)	1849 (14.5)	244 (18.8)	85 (12.7)	1492 (13.8)	329 (17.2)	357 (82.5)

NA = not applicable; Source: Brazilian National Health Survey, 2013.

**Table 2:** Prevalence of moderate and severe dependence in basic activities of daily living (BADL) and instrumental activities of daily living (IADL) according to sociodemographic characteristics in Brazilian older adults who participated in the Brazilian National Health Survey (2019 PNS).

	2019 PNS						
	BALD				IADL		
Variable	Total Population	Independent	Moderate	Severe	Independent	Moderate	Severe
	n = 22,728	n = 18,141	n = 3040	n = 1547	n = 2967	n = 14,354	n = 5407
**Chronic Disease**							
Diabetes or Hypertension	10,232 (47.5)	8053 (47.0)	1470 (51.4)	709 (45.0)	326 (13.0)	7193 (52.1)	2713 (51.5)
Both	3169 (15.2)	2176 (13.3)	628 (20.8)	365 (26.2)	37 (1.2)	1988 (15.3)	1144 (21.8)
None	8567 (37.3)	7283 (39.7)	853 (27.7)	431 (28.8)	2324 (85.8)	4827 (32.6)	1416 (26.7)
NA	760	629	89	42	280	346	134
**Age**							
60–69	12,555 (56.3)	10,756 (60.7)	1345 (44.9)	454 (28.7)	2154 (74.0)	8788 (62.6)	1613 (29.7)
70–79	7157 (30.1)	5584 (29.3)	1091 (35.2)	482 (29.5)	692 (21.8)	4469 (30.0)	1996(34.8)
80–89	2580 (11.5)	1652 (9.2)	508 (16.8)	420 (27.2)	115 (3.9)	1036 (7.1)	1429 (27.6)
90+	436 (2.1)	149 (0.8)	96 (3.1)	191 (14.6)	6 (0.3)	61 (0.3)	369 (7.9)
**Sex**							
Female	12,535 (56.7)	9654 (54.6)	1918 (64.5)	963 (64.6)	9654 (54.6)	1918 (64.5)	963 (64.6)
Male	10,193 (43.3)	8487 (45.4)	1122 (35.5)	584 (35.4)	8487 (45.4)	1122 (35.5)	584 (35.4)
**Race**							
White	9901 (50.5)	7948 (50.9)	1287 (48.0)	666 (50.7)	1155 (48.5)	6544 (52.0)	2202 (47.5)
Black	12,456 (47.7)	9890 (47.1)	1709 (50.9)	857 (47.6)	1755 (49.2)	7562 (46.1)	3139 (51.1)
Others	369 (1.8)	301 (1.9)	44 (1.2)	24 (1.7)	56 (2.2)	247 (1.9)	66 (1.3)
NA	2	0	0	0	1	1	0
**Marital Status**							
With partner	9946 (50.7)	8221 (52.6)	1233 (45.8)	492 (37.5)	1433 (55.4)	6726 (54.3)	1787 (38.2)
Without partner	12,782 (49.3)	9920 (47.4)	1807 (54.2)	1055 (62.5)	1534 (44.6)	7628 (45.7)	3620 (61.8)
**Education level**							
Until elementary school	14,987 (63.3)	11,445 (59.6)	2307 (76.5)	1235 (79.1)	1929 (58.6)	8612 (57.6)	4446 (81.4)
High school or more	7741 (36.7)	6696 (40.4)	733 (23.5)	312 (20.9)	1038 (41.4)	5742 (42.4)	961 (18.6)
**Region**							
Urban	17,313 (85.5)	13,750 (85.4)	2361 (86.2)	1202 (84.7)	2066 (81.6)	11,228 (87.3)	4019 (82.5)
Rural	5415 (14.5)	4391 (14.6)	679 (13.8)	345 (15.3)	901 (18.4)	3126 (12.7)	1388 (17.5)

NA = not applicable; Source: Brazilian National Health Survey, 2019.

**Table 3:** Association between diabetes and hypertension with moderate and severe BALD disability adjusted for education, sex, marital status, region, and age in Brazilian older adults, Brazilian National Health Survey 2013 and 2019.

BALD	2013 PNS				2019 PNS			
Variables	Moderate		Severe		Moderate		Severe	
	PrAdj (CI 95%)	*p* Value	PrAdj (CI95%)	*p* Value	PrAdj (CI 95%)	*p* Value	PrAdj (CI95%)	*p* Value
Chronic Disease								
Only one	1		1		1		1	
Both	1.39 (1.01–1.91)	0.04	1.45 (0.85–2.47)	0.16	1.20 (1.07–1.36)	<0.001	1.82 (1.59–2.07)	<0.001
None	0.70 (0.58–0.85)	<0.001	0.79 (0.51–1.11)	0.15	0.75 (0.66–0.84)	<0.001	0.92 (0.82–1.04)	0.20
Elementary vs. High school or more	1.64 (1.29–2.11)	<0.001	1.63 (1.15–4.02)	0.005	1.72 (1.56–1.91)	<0.001	1.52 (1.32–1.74)	<0.001
Female vs. Male	0.97 (0.80–1.18)	0.82	1.01 (0.65–1.56)	0.95	1.29 (1.19–1.40)	<0.001	1.08(0.95–1.24)	0.21
Not married vs. married	1.05 (0.85–1.30)	0.60	1.73(1.06–2.83)	0.02	1.00 (0.91–1.09)	0.94	1.27 (1.11–1.45)	<0.001
Urban vs. rural	0.77 (0.62–0.95)	0.01	1.11 (0.70–1.76)	0.63	1.15 (1.14–1.45)	0.01	0.96 (0.86–1.07)	0.54
Age (in years)								
60–69	1		1		1		1	
70–79	1.27 (1.03–1.57)	0.02	2.53 (1.59–4.02)	<0.001	1.28 (0.89–0.90)	<0.001	1.69 (1.46–1.96)	<0.001
80–89	1.99 (1.58–2.52)	<0.001	5.63 (3.36–9.43)	<0.001	1.58 (1.39–1.79)	<0.001	3.84 (3.26–4.53)	<0.001
90+	2.47 (1.78–3.43)	<0.001	10.41 (6.68–16.21)	<0.001	1.56 (1.22–1.99)	<0.001	11.1 (9.64–12.8)	<0.001

PrAdj: Adjusted Prevalence Ratio; 95% CI: 95% Confidence Interval; Source: Brazilian National Health Survey, 2013 and 2019.

**Table 4:** Adjusted analysis of the association between chronic diseases and moderate and severe dependence in Brazilian older adults in IADL, National Health Survey 2013 and 2019.

IADL	PNS 2013				PNS 2019			
Variables	Moderate		Severe		Moderate		Severe	
	PrAdj (95%CI)	*p* Value	PrAdj (95%CI)	*p* Value	PrAdj (95%CI)	*p* Value	PrAdj (95%CI)	*p* Value
Chronic Disease								
Only one	1	1	1	1	1	1	1	1
Both	1.03 (0.83–1.28)	0.73	1.23 (1.05–1.44)	0.008	0.92 (0.91–0.93)	<0.001	1.29 (1.23–1.34)	<0.001
None	0.89 (0.75–1.05)	0.18	0.79 (0.69–0.90)	0.006	0.76 (0.75–0.77)	<0.001	0.77 (0.73–0.81)	<0.001
Female vs. Male	0.97 (0.82–1.14)	0.71	1.32 (1.14–1.53)	<0.001	0.96 (0.95–0.97)	<0.001	1.34 (1.27–1.42)	<0.001
Urban vs. Rural	0.96 (0.79–1.16)	0.69	0.89 (0.79–1.00)	0.055	1.08 (1.07–1.10)	<0.001	0.87(0.84–0.90)	<0.001
Elementary vs. High school or more	2.00 (1.64–2.44)	<0.001	2.04 (1.77–2.37)	<0.001	0.85 (0.85–0.86)	<0.001	1.82 (1.71–1.94)	<0.001
Not married vs. married	1.32 (1.10–1.60)	0.003	1.22(1.06–1.41)	0.005	0.93 (0.92–0.94)	<0.001	1.19 (1.14–1.26)	<0.001
Black race	0.92 (0.79–1.08)	0.22	1.14 (1.00–1.29)	0.04	0.95 (0.94–0.96)	<0.001	1.06 (1.03–1.10)	<0.001
Other races	0.79 (0.54–0.1.15)	0.35	0.65 (0.45–0.92)	0.01	0.98 (0.94–1.01)	0.24	0.73 (0.58–0.98)	0.04
Age (in years)								
60–69	1		1		1		1	
70–79	1.71 (1.42–2.07)	<0.001	2.28 (1.91–2.72)	<0.001	0.90 (0.88–0.90)	<0.001	1.88 (1.76–2.00)	<0.001
80–89	1.88 (1.52–2.32)	<0.001	5.07 (4.33–5.94)	<0.001	0.57 (0.55–0.59)	<0.001	3.69 (3.47–3.92)	<0.001
90+	1.34 (0.75–2.39)	0.30	8.52 (7.36–9.87)	<0.001	0.12 (0.10–0.15)	<0.001	5.65 (5.32–6.00)	<0.001

PrAdj: Adjusted Prevalence Ratio; 95% CI: 95% Confidence Interval; Source: Brazilian National Health Survey, 2013 and 2019.

## Data Availability

The PNS is a national household-based survey where data are made available openly and free of charge to any individual. The data are available in a publicly accessible repository. Since our study was conducted with these secondary data, the repository of the two databases can be found at the link [https://www.ibge.gov.br/estatisticas/sociais/saude/9160-pesquisa-nacional-de-saude.html?=&t=downloads] (accessed on 27 May 2025).

## Abbreviations

The following abbreviations are used in this manuscript:

95%CI	95% Confidence Interval
BADL	Basic Activities of Daily Living
DALY	Disability-Adjusted Life Years
GBD	Global Burden of Disease
IADL	Instrumental Activities of Daily Living
IBGE	Instituto Brasileiro de Geografia e Estatística
NCDs	Chronic noncommunicable diseases
NHS	National Health Survey
PNS	Pesquisa Nacional de Saúde
PRa	Prevalence-Ratio-Adjusted (ou conforme sua definição específica)
Pw	Probability weight
SAH	Systemic Arterial Hypertension
T2DM	Type 2 Diabetes Mellitus

## Appendix A

**Table A1:** Definition of the operational study adjustment variables in the baseline and the new categorizations.

Variable	Definitions in the Original Study	New Categorizations
Age	In years	In years
Sex	Male	Masculine
	Female	Feminine
Education	Uneducated	Incomplete elementary education
	Incomplete elementary school	
	Complete elementary	Completed elementary education
	Incomplete medium	
	Complete medium	
	Incomplete higher education	
	Completed higher education	
Race	White	White
	Yellow	Black
	Brown	Others
	Black	
	Indigenous	
Region	Rural	Rural
	Urban	Urban
Marital status	Married	Married
	Divorced	Not married
	Separate	
	Single	
	Widower	
Type 2 Diabetes Mellitus	Self-reported medical diagnosis	0—does not have diabetes
		1—has diabetes
Systemic Arterial Hypertension	Self-reported medical diagnosis	0—no hypertension
		1—has hypertension
	No difficulty	0—cannot, has much difficulty, and has little difficulty
	There is little difficulty	
	It has great difficulty	1—No difficulty
	Cannot	
IADL	No difficulty	0—Cannot, has much difficulty, and has little difficulty
	There is little difficulty	
	It has great difficulty	1—No difficulty
	Cannot	

**Table A2:** Adherence of this study to the STROBE criteria.

Item No.	STROBE Recommendation	Location in the Manuscript (Page, Paragraph)
1(a)	Indicate the study’s design with a commonly used term in the title or the abstract	Title (p. 1, 1)
1(b)	Provide in the abstract an informative and balanced summary of what was carried out and what was found	Abstract (p. 1,1)
2	Explain the scientific background and rationale for the investigation being reported	Introduction (p. 1, 1–4)
3	State-specific objectives, including any prespecified hypotheses	Final of the introduction (p. 2, 4)
4	Present key elements of the study design early in the paper	Materials and Methods (p. 2, 1–2)
5	Describe the setting, locations, and relevant dates, including periods of recruitment, exposure, follow-up, and data collection	Materials and Methods (p. 3, 3)
6(a)	Give the eligibility criteria, and the sources and methods of selection of participants	Materials and Methods (p. 3, 4)
7	Clearly define all outcomes, exposures, predictors, potential confounders, and effect modifiers. Give diagnostic criteria, if applicable	Materials and Methods (p. 3, 4–6)
8	For each variable of interest, give sources of data and details of methods of assessment (measurement). Describe the comparability of assessment methods if there is more than one group	Materials and Methods (p. 3, 5–6)
9	Describe any efforts to address potential sources of bias	Does not apply
10	Explain how the study size was arrived at	Materials and Methods (p. 2, 2)
11	Explain how quantitative variables were handled in the analyses. If applicable, describe which groupings were chosen and why	Materials and Methods (p. 3, 5–6)
12(a)	Describe all statistical methods, including those used to control for confounding	Materials and Methods (p. 3, 7)
12(b)	Describe any methods used to examine subgroups and interactions	Does not apply
12(c)	Explain how missing data were addressed	Materials and Methods (p. 3, 5)
12(d)	If applicable, describe analytical methods taking account of sampling strategy	Materials and Methods (p. 2, 7)
12(e)	Describe any sensitivity analyses	Does not apply
13(a)	Report numbers of individuals at each stage of study (e.g., potentially eligible, examined for eligibility, included, analyzed)	Results (p. 4, 1)
13(b)	Give reasons for non-participation at each stage	Results (p. 4, 1)
13(c)	Consider use of a flow diagram	Results (p. 4, 1)
14(a)	Give characteristics of study participants (e.g., demographic, clinical, and social) and information on exposures and potential confounders	Results (p. 6 and 8)
14(b)	Indicate number of participants with missing data for each variable of interest	Results (p. 6 and 8)
15	Report numbers of outcome events or summary measures	Results (p. 6 and 8)
16(a)	Give unadjusted estimates and, if applicable, confounder-adjusted estimates and their precision (e.g., 95% confidence interval). Make clear which confounders were adjusted for and why they were included	Results (3 and Table 4)
16(b)	Report category boundaries when continuous variables were categorized	Methods (p. 3, 4)
16(c)	If relevant, consider translating estimates of relative risk into absolute risk for a meaningful time period	Does not apply
17	Report other analyses completed, e.g., subgroup analyses and sensitivity analyses	Does not apply
18	Summarise key results with reference to study objectives	Discussion (p. 13)
19	Discuss limitations of the study, taking into account sources of potential bias or imprecision	Discussion (p. 13, 4–5)
20	Give a cautious overall interpretation of results considering objectives, limitations, multiplicity of analyses, results from similar studies, and other relevant evidence	Discussion and conclusions (p. 13, 5–6; p. 14, 4)
21	Discuss the generalizability (external validity) of the study results	Conclusions (p. 14, 1)
22	Give the source of funding and the role of the funders for the present study	p. 15, 2

**Table A3:** Descriptive table of raw Basic Activities of Daily Living (BADL) data in 2013 PNS.

Categories	K001 To Eat	K004 To Bath	K007 Bathroom Use	K010 To Dress Up	K013 To Walk	K016 + K019 Transfer
No difficulty	10,667 (95.44)	10,469 (93.67%)	10,536 (94.27%)	10,239 (91.61%)	10,290 (92.06%)	20,573 (92.03)
There is little difficulty	316 (2.83)	354 (3.17)	352 (3.15)	551 (4.93)	522 (4.67)	1161 (5.19)
It has great difficulty	125 (1.12%)	180 (1.61%)	139 (1.24%)	215 (1.92%)	222 (1.99%)	360 (1.61%)
Cannot	69 (0.62%)	174 (1.56%)	150 (1.34%)	172 (1.54%)	143 (1.28%)	260 (1.16%)

**Table A4:** Description of raw data on Instrumental Activities of Daily Living (IADL) in 2013 PNS.

Categories	K022 Shopping	K025 Finances	K028 Medicines	K031 Doctor	K034 Transport
No difficulty	9302 (83.22%)	9891 (88.49%)	8211 (73.46%)	8646 (77.36%)	8732 (78.12%)
There is little difficulty	678 (6.07%)	478 (4.28%)	391 (3.50%)	1151 (10.30%)	921 (8.24%)
It has great difficulty	457 (4.09%)	263 (2.35%)	205 (1.83%)	630 (5.64%)	639 (5.72%)
Cannot	740 (6.62%)	545 (4.88%)	245 (2.19%)	750 (6.71%)	885 (7.92%)
Does not use medication	-	-	2125 (19.01%)	-	-

**Table A5:** Description of raw Basic Activities of Daily Living (BADL) data in 2019 PNS.

Categories	K001 To Eat	K004 To Bath	K007 Bathroom Use	K010 To Dress Up	K013 To Walk	K016 + K019 Transfer
No difficulty	21,613 (95.09%)	21,061 (92.67%)	20,681 (90.99%)	20,052 (88.23%)	20,289 (89.27%)	39,898 (87.77%)
There is little difficulty	697 (3.07%)	936 (4.12%)	1086 (4.78%)	1749 (7.70%)	1405 (6.18%)	3576 (7.87%)
It has great difficulty	250 (1.10%)	374 (1.65%)	372 (1.64%)	590 (2.60%)	517 (2.27%)	1.015 (2.23%)
Cannot	168 (0.74%)	357 (1.57%)	589 (2.59%)	337 (1.48%)	517 (2.27%)	967 (2.13%)

**Table A6:** Description of raw data on Instrumental Activities of Daily Living (IADL) in 2019 PNS.

Categories	K022 Shopping	K025 Finances	K028 Remedy	K031 Doctor	K034 Transport
No difficulty	18,473 (81.28%)	19,722 (86.77%)	602 (2.65%)	16,956 (74.60%)	16,916 (74.43%)
There is little difficulty	1768 (7.78%)	1293 (5.69%)	482 (2.12%)	2578 (11.34%)	2383 (10.48%)
It has great difficulty	963 (4.24%)	607 (2.67%)	1295 (5.70%)	1226 (5.39%)	1367 (6.01%)
Cannot	1524 (6.71%)	1106 (4.87%)	16896 (74.34%)	1968 (8.66%)	2062 (9.07%)
Does not use medication	-	-	3453 (15.19%)	-	-
